# A Novel Anti-Tumor Inhibitor Identified by Virtual Screen with PLK1 Structure and Zebrafish Assay

**DOI:** 10.1371/journal.pone.0053317

**Published:** 2013-04-26

**Authors:** Jing Lu, Shengchang Xin, Huan Meng, Matt Veldman, David Schoenfeld, Chao Che, Ruibin Yan, Hanbing Zhong, Song Li, Shuo Lin

**Affiliations:** 1 Shenzhen Graduate School of Peking University, Shenzhen, China; 2 Department of Molecular, Cell and Developmental Biology, University of California Los Angeles, Los Angeles, California, United States of America; Johns Hopkins School of Medicine, United States of America

## Abstract

Polo-like kinase 1 (PLK1), one of the key regulators of mitosis, is a target for cancer therapy due to its abnormally high activity in several tumors. Plk1 is highly conserved and shares a nearly identical 3-D structure between zebrafish and humans. The initial 10 mitoses of zebrafish embryonic cleavages occur every∼30 minutes, and therefore provide a rapid assay to evaluate mitosis inhibitors including those targeting Plk1. To increase efficiency and specificity, we first performed a computational virtual screen of∼60000 compounds against the human Plk1 3-D structure docked to both its kinase and Polo box domain. 370 candidates with the top free-energy scores were subjected to zebrafish assay and 3 were shown to inhibit cell division. Compared to general screen for compounds inhibiting zebrafish embryonic cleavage, computation increased the efficiency by 11 folds. One of the 3 compounds, named I2, was further demonstrated to effectively inhibit multiple tumor cell proliferation *in vitro* and PC3 prostate cancer growth in Xenograft mouse model *in vivo*. Furthermore, I2 inhibited Plk1 enzyme activity in a dose dependent manner. The IC_50_ values of I2 in these assays are compatible to those of ON-01910, a Plk1 inhibitor currently in Phase III clinic trials. Our studies demonstrate that zebrafish assays coupled with computational screening significantly improves the efficiency of identifying specific regulators of biological targets. The PLK1 inhibitor I2, and its analogs, may have potential in cancer therapeutics.

## Introduction

The PLK family of serine threonine kinases is characterized by their C-terminal polo-box domains (PBD), which function to interact with other proteins to maintain region-specific localization within the cell [1], [2]. Following the original discovery of the PLK family in *D. melanogaster* [3], [4], PLKs have been found in all animals and fungi examined to date. In mammals, 5 different homologs of PLK have been identified: PLK1, PLK2 (SNK), PLK3 (PRK), PLK4 (SAK) [5]and PLK5[6]. In mice, gene knockouts of PLK1 [7] and PLK4 [8] were not viable, while PLK2 [9] null mice exhibit dwarfism and PLK3 [10] null mice became susceptible to tumors. Haploinsufficiency of PLK1/4 has also been reported in mice, both leading to an increased incidence of tumors [7]–[11]. Thus far, PLK1 and its non-mammalian orthologs has been the most extensively characterized member for its regulatory role in several vital steps of mitosis and cytokinesis. PLK1 is known to promote M phase via cyclin/Cdk phosphorylation [12]–[14], coordinate bipolar spindle formation [15], promote sister chromatid separation [16], modulate anaphase promoting complex activity for mitotic exit [17], and play a role in the appropriate localization of RhoA for cytokinesis [18]. PLK4 has also been determined to be a crucial factor in the duplication of centrioles [19], [20]. While the functions of PLK2/3 have remained largely unexplored, PLK3 has been implicated in promotion of S-phase [21] and DNA-damage repair [22], [23].

Overexpression of PLK1 has been found in the genetic profile of several different human cancers, and has been linked with poor prognosis [24]–[26]. PLK1 has also been implicated in the suppression of p53 apoptotic activity, in addition to PLK1's other proliferative functions [27]. Depletion of PLK1 in cancer cells *in vitro* has been demonstrated to inhibit proliferation [28]–[31]. Furthermore, normal cells appear to have a higher degree of resilience to reductions in PLK1 expression [32], making it a highly desirable target for chemotherapy with several compounds already in phase I clinical trials [33]–[35]. However, while other PLK family members are known to have functions independent of PLK1 activity, those functions are not yet fully understood. Given the structural similarity of the kinase and polo-box domains between the PLKs, finding additional inhibitory compounds specific to PLK1 or to a smaller subset of the PLK family would be of great utility in developing therapeutic applications.

In the effort to evaluate the efficacy of PLK1 inhibitors, zebrafish have proven to be an excellent model for investigation. Zebrafish possess a PLK1 ortholog, as well as PLK2a, PLK2b, PLK3 and PLK4 [36]. Inhibition of PLK1 by morpholino injection into zebrafish embryos (morphant) demonstrated dose-dependent cell death in proliferating tissues, as well as centrosome instability, impaired spindle assembly, and aneuploidy resulting from failed chromosome separation [37]. By comparing phenotypes induced by different chemical compounds to that of PLK1 zebrafish morphant, the activity and specificity of potential Plk1 chemical inhibitors can be determined *in vivo*.

Although impressive in terms of screening capacity as a vertebrate model, zebrafish embryonic assays still cannot match the scale and speed of enzymatic or cell based assays. To overcome this limitation, we have adopted computation as a strategy to pre-select candidate compounds for further zebrafish analysis. Positive hits from the virtual screen can then be screened using intact zebrafish embryos. In this study, using computational analysis of possible interactions of approximately 60000 compounds with the kinase and polo-box domains of PLK1, 370 compounds were selected as possible candidates for high binding affinity. These candidate compounds were analyzed for their ability to inhibit cell division of early embryos. As described below, this approach has successfully led to the identification of one novel compound that has promising potential as a PLK1 inhibitor with anti-tumorigenic activity.

## Results

### Virtual Screening

**Figure 1:**
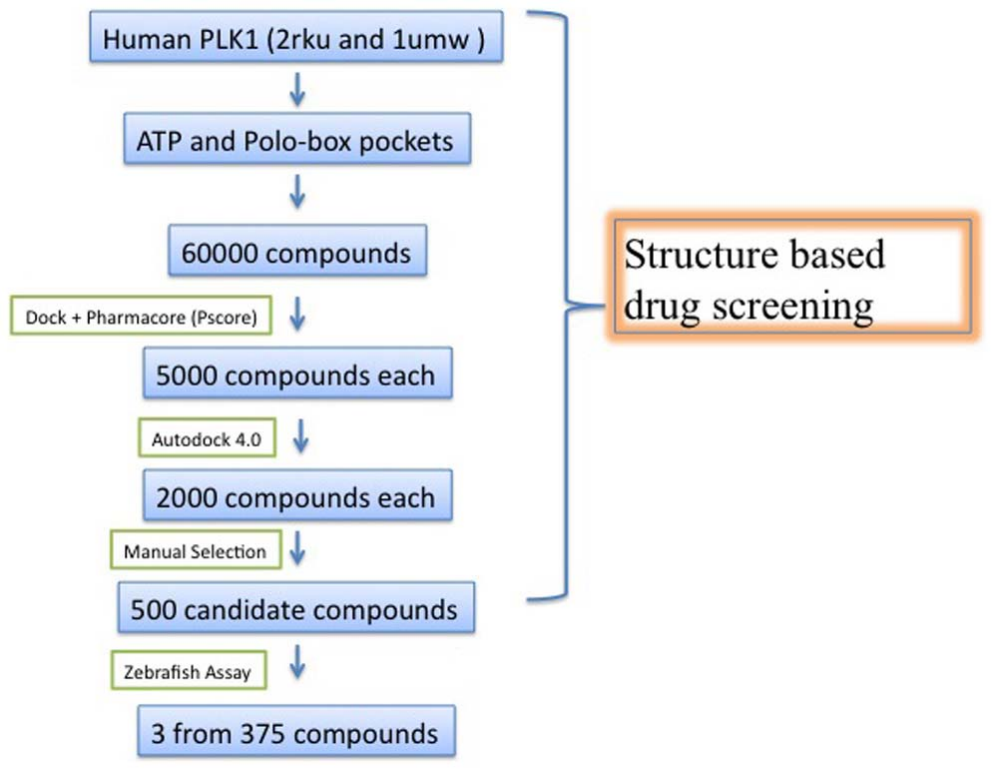
Overall screening strategy for Plk1 inhibitor.

Our strategy (Fig. 1) to discover Plk1 inhibitors started from rapid rigid docking using the program DOCK coupled with pharmcophore filtering program Pocket v.2. The two computational procedures reduced the candidate compounds from 60,000 to 5,000 based on binding energy of kinase domain and PBD. Autodock4.0 was then used to further select better candidates. After this step, the top 2000 compounds ranked by energy-score in autodock4.0 were manually checked for binding conformation. This step generated 370 candidates that had the best conformation match to Plk1 kinase domain and PBD. These compounds were then selected for *in vivo* screening in zebrafish.

### Zebrafish Screening

Zebrafish embryos develop extremely rapidly and are highly accessible for direct microscopic observation. Several previous studies have utilized these advantages and established that zebrafish embryos as a useful whole animal screening model for the activity of small chemical compounds [38]. During early development, cell division is very active, with cells completing entire cycles in as little as 20 minutes. Genetic studies have demonstrated that Plk1 function is highly conserved across all tested animals and fungi and inhibition of Plk1 by small molecules results in the immediate ceasing of cleavage of early embryos undergoing mitosis. We therefore expect that some of our compounds with high affinity scores to Plk1 by computation should have an inhibitory effect on the early cleavage of zebrafish embryos.

**Figure 2:**
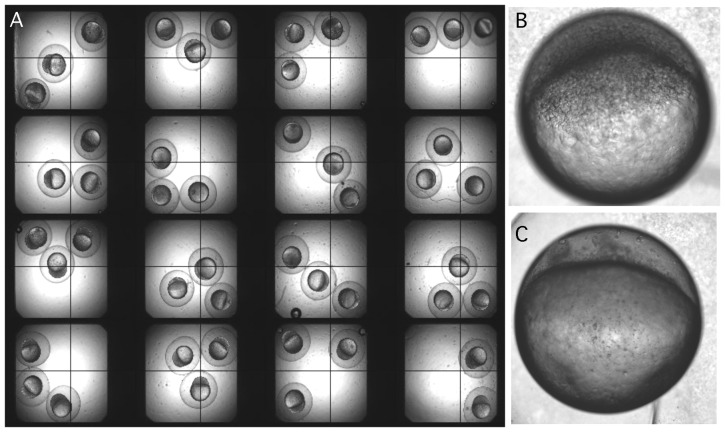
Screening for mitotic inhibitor using zebrafish embryo. A. Embryos were incubated with compounds in multi well plates and visualized under a dissection microscope. A compound was considered as positive if division of all three embryos in the well was inhibited. B. A normal embryo after 4 hours of development. C. An embryo with cell division inhibited at 1 cell stage when an active inhibitor was present even after 4 hours of development.

After selecting 370 compounds by their ranking of free-energy scores, each compound was individually screened against zebrafish embryonic cleavage. After duplicating screens, 3 compounds were shown to inhibit cell division within 40 minutes after addition to the embryos (Figure 2). Titration analysis by 2×dilution from initial 10 mM working solution showed that the effective concentrations for these compounds were in the range of 0.5–2.0 uM, similar to that of the positive control of ON-01910, a non-ATP-competitive small molecule inhibitor of Plk1 with potent anti-proliferative activity.

To determine if computation based pre-screen increased the efficiency of identifying inhibitors of mitosis, we concurrently performed a random screen using the chemical library of ChemBridge DiverSet. To date, we have completed screening of 5376 compounds and 4 were confirmed to stop cell division when added at 2–4 cell stage embryos. This finding suggests that our pre-screen increased the efficiency of identifying mitosis inhibitors by approximately 11 fold.

### I2 inhibits in vitro proliferation of multiple human tumor cells

**Figure 3:**
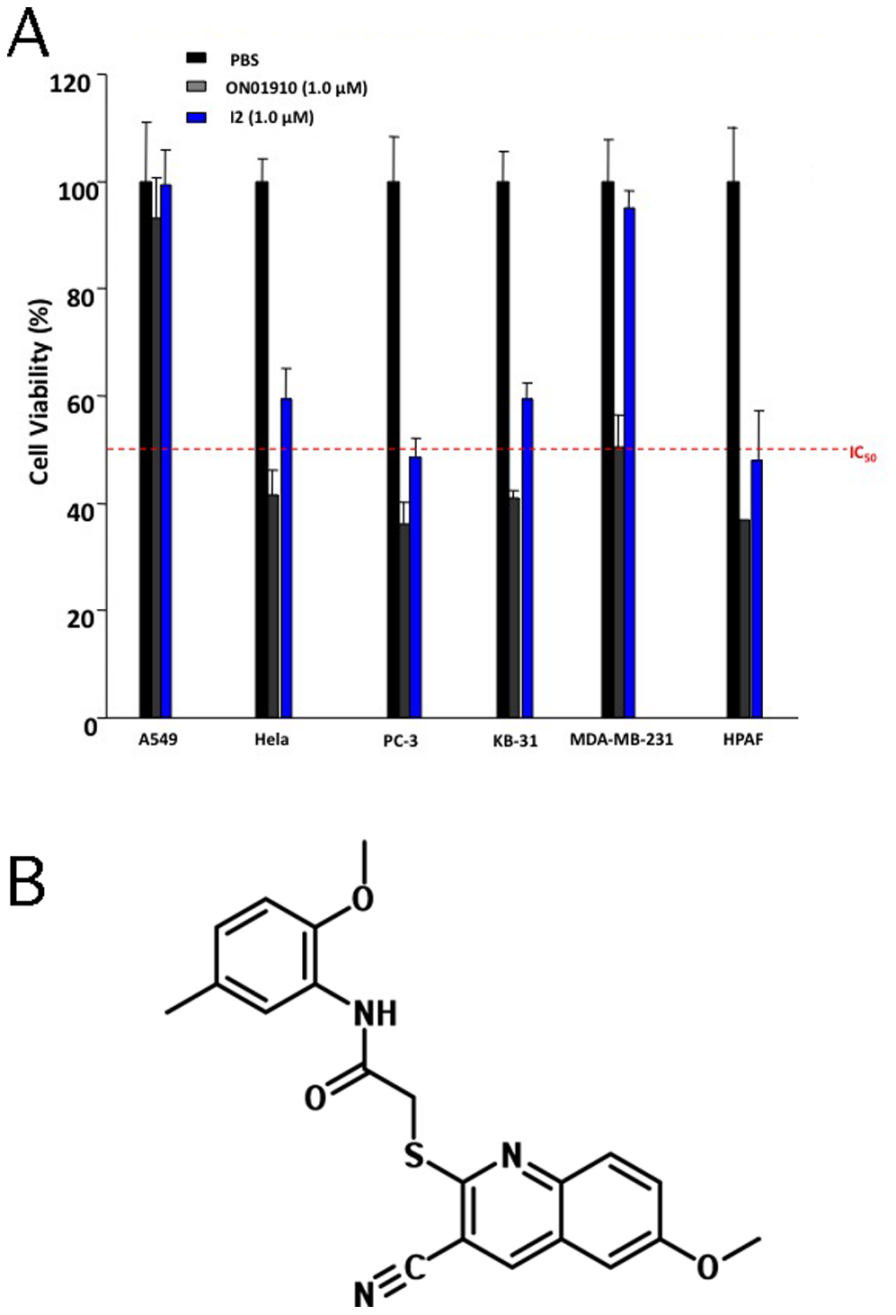
Tumor cell proliferation inhibited by I2 and its chemical structure. A. Comparison of inhibition of tumor cell viability by I2 and ON01910 relative to PBS control. B. Structure of I2.

We then performed an independent cell proliferation assay by treating five cancer cell lines with various concentrations of the compounds capable of inhibiting zebrafish embryonic cleavage. We found that only one of the three, named I2 (Figure 3), showed an efficacy of inhibiting three human tumor cell proliferation with potency similar to that of ON-01910 (Figure 3). We next expanded the test of I2 on additional ten tumor cells and confirmed its broad ability to inhibit multiple tumor cell proliferation, although with variable efficiency (Figure S1in File S1). I2 showed equivalent inhibitory effect on these cancer cells compared to ON01910. It is worth noting that IC_50_ of I2 in JF-305, HCT116 and COLO-205 were less than 1 µM. Chemical informatics analysis suggested that I2 represents a new compound that had not been previously studied in any biological assays. I2 was therefore selected for further experiments to identify its active analogs, and to determine its ability to inhibit tumor growth *in vivo* and PLK1 enzymatic activity.

### Analysis of I2 analogs: structure activity relationship to mitosis inhibition

We purchased 12 I2 analogs from commercial venders and also synthesized an additional 20 analogs based I2 core structure (Table S1 in File S1). Rapid assay of their ability to inhibit mitotic cell division of zebrafish embryos was then performed. In addition to I2, the compounds named A3 and 5d showed inhibition activity of zebrafish embryonic cleavage.

The structure-activity relationships (SARs) of I2 analogs were investigated in quinoline and anilide templates. In the quinoline template, the replacement of methoxyl in the 6-position with ethoxyl, ethyl or methyl caused a loss of activity. Likewise, halide groups also were not tolerated in this position. Compounds with Br or Cl substitution in the 6 position brought about the loss in activity, highlighting the essential role of 6-methoxyl in the scaffold. In the anilide template, 2-methoxyl-5-methyl was the preferred substituent pattern. However, fine modification in these two positions did not make any improvement, and even drastically decreased the activity. For example, 2,5-dimethoxyl and 2-methoxyl-5-Cl analogs lost their potency. However, substitution in 2-position was well tolerated, and 2-trifluoromethyl analog (A3) had comparable activity (2 µM) to the parent I2, whereas compounds with 2-Br exhibited a decreased activities at 50 µM. Variation of substituents in other positions or changing the phenyl to heterocycles such as pyridine or pyrazine also resulted in the loss of potency, suggesting that SARs here are relatively restricted.

For all 32 analogues, we independently compared their ability to inhibit tumor cell proliferation *in vitro* with I2 and noted that I2 still appeared to be the most potent one (Table S1in File S1). Therefore, we kept I2 as the top candidate for further biological studies.

### I2 suppresses ectopically implanted PC-3 and HCT116 tumor xenograft growth

**Figure 4:**
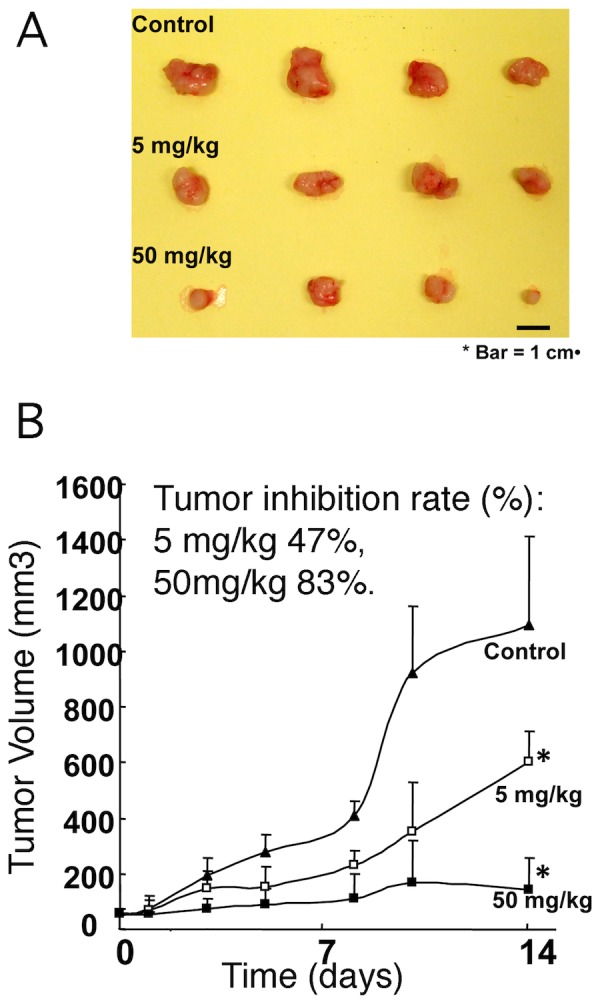
PC3 tumor growth inhibited by I2 *in vivo*. After treatment with I2 as described in material and method, tumors were dissected out, measured and imaged. The inhibition rate of two doses of I2 (B) was consistent with size of tumors as shown in (A).

Figure 4A and B shows the effect of I2 treatment on growth of PC-3 tumor xenografts in nude mice. A statistically significant inhibition (P<0.05) of PC-3 xenograft growth was observed when 5 mg/kg was given to the mice intraperitoneally (Fig. 4B). On day 14, the average tumor weight in control mice was approximately 47% higher compared with I2 (5 mg/kg) treated mice. These results indicated that the growth of PC-3 xenograft in nude mice was retarded upon I2 administration. The body weights of the control and treated mice were determined periodically to assess non-specific toxicity of I2. When 5 mg/kg of I2 was given, the average body weights of the control and I2 treated mice did not differ significantly, suggesting that I2 administration did not cause weight loss. However, when we increased the dose to 50 mg/kg, although the mice in I2 treated group increased tumor inhibition rate to 83%, they showed about 30% weight loss, yet no death was observed after 14 days treatment. Based on this finding, we next tested I2′s ability to inhibit growth of colon cancer cell HCT116 in xenografts at doses of 5 mg/kg, 25 mg/kg and 37.5 mg/kg. As shown in supplementary materials, HCT116 growth *in vivo* was inhibited in a dose dependent manner showing approximately 14% 41% and 53% tumor reduction, respectively (Figure S2 in File S1). Under these conditions no weight loss was observed. Overall, these studies suggest that I2 is reasonably tolerated by mice and is capable of inhibiting tumor growth *in vivo*.

### I2 induces zebrafish phenotype similar to Plk1 deficiency, inhibits mitotic chromosomal segregation and Plk1 enzyme activity

**Figure 5:**
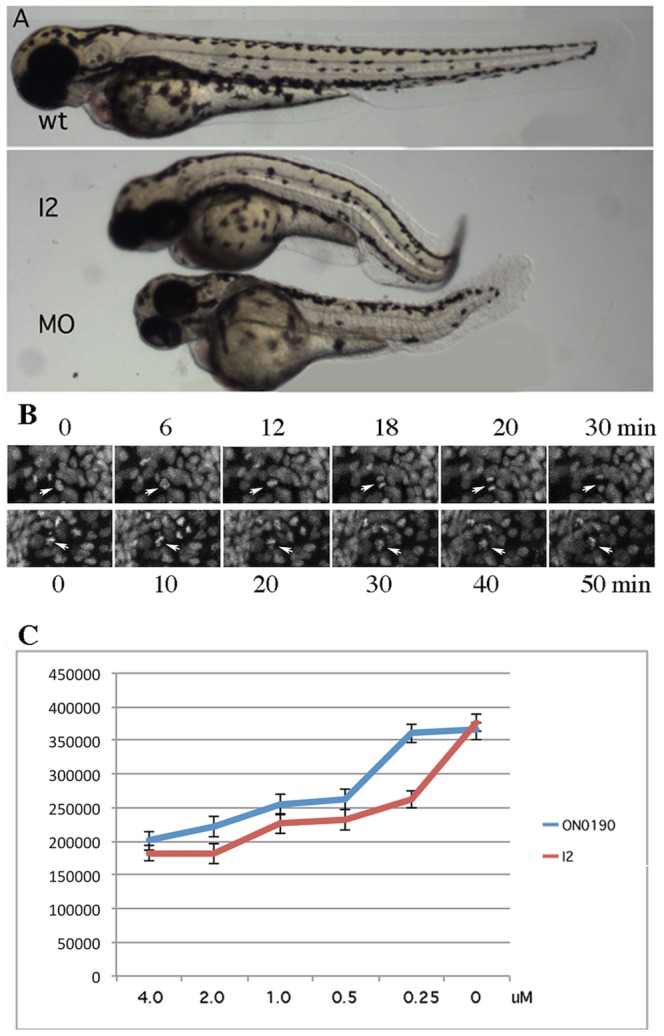
Analysis of I2 specificity against PLK1. A. Comparison of zebrafish embryonic phenotype induced by I2 inhibition and Plk1 morpholino. Wild type embryo (upper) developed normally whereas I2 treated embryos (middle) and Plk1 morpholino injected embryo (lower) were shorter and appeared similarly deformed. B. Inhibition of chromosomal segregation in live zebrafish embryos. H2A GFP labeled chromosomes in wild type controls segregated in approximately 15 minutes (arrows, the upper panel) while the chromosomes (arrows, lower panel) of I2 treated embryo attempted to segregate but failed to do so after 50 minutes. C. Inhibition of PLK1 enzyme activity by I2 and ON01910. The values of three independent assays were averaged.

To confirm that I2 has specific inhibitory effect on Plk1 activity, three approaches were employed. First, as previously published, zebrafish embryos showed a specific phenotype when Plk1 is knocked down by its specific antisense morpholino (5). We compared the phenotype of Plk1 morphant with embryos treated with I2 at late gastrulation stage and found that the overall morphological phenotypes were very similar to each other (Figure 5A). The Plk1 morphant (embryos injected with plk1 anti-sense morpholino oligos) and embryos treated with I2 both displayed a phenotype of smaller heads, shorter bodies and curling tails. Secondly, H2A-GFP transgenic embryos, which were used to facilitate the monitoring of chromosome movements, showed delayed or stopped chromosome segregation upon treatment of I2 (Figure 5B and Table S2 in File S1). In average, cell division takes about 16 minutes to finish in normal embryos while I2 treatment delayed the time beyond 50 minutes. In addition, I2 treatment reduced cells undergoing mitosis from∼20% to 8% at the embryonic stage of observation. This observation is very similar to the published finding of inhibiting Plk1 in zebrafish by MO or another chemical inhibitor BI 2536[39]. Finally, to obtain more direct evidence, we performed an *in vitro* kinase assay to determine if I2 could inhibit pure PLK1's phosphoryllation of its recombinant substrate casein. As seen in Figure 5C, PLK1 activity was inhibited by I2 in a dose dependent manner with an inclination similar to that of ON-01910, suggesting a higher but comparable IC_50_. Overall, these assays suggest that I2 is likely a candidate for inhibition of PLK1.

### I2 arrests cell cycle at G0/G1 phase and increases apoptosis

To determine I2′s effect on cell cycle progression cellular DNA content of PC-3 was analyzed by flow cytometry. The I2-treated cells showed a significant block of cell cycle progression at G0/G1 phase and increased the percentage of cells in G0/G1 phase from 7% to 40% and decreased S phase from 43% to 28% (Figure S3 in File S1). In addition, treatment of I2 on zebrafish embryos also induced significant increase of apoptotic cells (Figure S3 in File S1). These findings indicated that I2 acts at the early events of the cell cycle to be effective against DNA synthesis and consequently cause apoptosis in treated cells.

## Discussion

Zebrafish has been established as an excellent vertebrate system for screening pharmaceutical drugs or biological active small molecules. However, its throughput in such applications is somewhat limited by the availability of large number of embryos and relatively slow process of handling and imaging whole embryos. Here we developed an effective screening approach for mitosis inhibitors by combining computation virtual analysis and rapid zebrafish embryo assay. In general, incorporation of a pre-screen by computation reduces the sample numbers and therefore expands the efficiency of the whole organism based screen as well as increases the specificity of target interaction. The specific mitosis inhibitor I2 we identified from this screen is likely a PLK1 inhibitor since we used PLK1 protein structures as the initial virtual screen target. We have also shown that it induced zebrafish embryonic phenotype similar to that of the Plk1 morphant, and blocked chromosomal segregation. It has an inhibitory activity against PLK1 enzyme with a similar inclination to ON-01910, a Phase III drug candidate that is characterized as a non-ATP-competitive PLK1 inhibitor. We have not determined if I2 acts as an ATP competitive or none competitive inhibitor. It might have dual actions since we identified it as a compound that has relatively high binding affinity to both ATP and polo box domains of human PLK1. Further experiments are needed to address this issue. Additionally, we cannot exclude the possibility that I2 has other targets including additional kinases since we have not performed an extensive profiling analysis against a large number of kinases. Nevertheless, this compound has anti tumor activity both *in vitro* and *in vivo*. Compared to previously published data, we noted that ON-01910 had lower activity of inhibiting tumor cell growth and Plk1 enzymatic activity in our experiments. We are not aware of any other independently reported activity data for ON-01910 but we repeatedly obtained comparable results between I2 and ON-01910 in our hand. I2 is a novel compound that is not commercially available in large quantities. With the synthesis approach described here, we are able to produce this compound efficiently, allowing further pharmacological studies of this compound.

## Materials and Methods

### Virtual screening

Five small molecule libraries were used: (1) FDA Approved Drug Library-a collection of 1120 high-purity chemical compounds carefully selected for structural diversity and broad spectrum covering several therapeutic areas, (2) Microsource Spectrum Collection-2000 biologically active and structurally diverse compounds from known drugs, experimental bioactives, and pure natural products, (3) Druggable Compound Set-A set of about 8000 compounds which are targeted at kinases, protease, ion channels and GPCR's, (4) Lead Like Compound Set-20,000 compounds custom-tailored for lead likeness, (5) ChemBridge DiverSet-30,000 chemically diverse small molecules. Approximately 60,000 compounds from these libraries were converted to 3D mol2 format and then used to perform structure-based drug screening. To select compounds that may bind to both the PLK1 kinase domain and PBD, the complex X-ray structure of human PLK1 kinase domain, along with the small molecular BI-2536 (PBD ID: 2RKU) and human PLK1 PBD-phosphopeptide complex (PDB ID: 1UMW) were each used to create possible conformations with individual small molecules by DOCK. After docking, Pocket v.2, a program developed based on protein structures without human intervention, was utilized as a pharmcophore filtering step to minimize false positive candidates. Autodock4.0 was then used to further select better candidates. Finally, manual inspection of binding conformation was implemented to select best candidates that may have higher affinity to both the PLK1 kinase domain and PBD.

### Zebrafish screening

Wild-type AB zebrafish were raised under standard conditions. The 370 compounds identified via computational analysis for Plk1 interaction were individually placed in 96-well plates with 200 µL of egg water containing a 10 µM solution of either the experimental compounds or On-01910 positive control. Embryos at the 2–4 cell stages were transferred to the plates (three embryos per well) and incubated at 28.5° for 1–2 hours. Embryos were then observed via a Zeiss Stemi 2000-C dissection microscope for the resulting phenotype. Similarly, a screen of approximately 5000 random compounds without pre-computational selection was carried out to determine efficiency of virtual screen. After positive compounds were identified, the minimal concentration for inhibition of zebrafish embryo cleavage was determined by treating embryos with 2×dilutions of 10 µM solutions.

### I2 and its analog synthesis

The synthetic details are described in the supplementary materials, and supplementary Scheme 1 in File S1, which illustrate the general synthetic route of I2 and its analogs. Starting from acetamide **1**, the 2-chloroquinoline-3-carbaldehyde **2** was obtained in moderate to high yield through Vilsmeier-Haack reaction. Reaction of compound **2** with hydroxylamine hydrochloride in dimethyl sulphoxide delivered 2-chloroquinoline-3-carbonitrile **3** which was then reacted with sodium hydrosulfide in refluxing ethyl alcohol to afford the corresponding thienoquinoline derivative **4**. Finally, the coupling of compound **4** with a variety of bromoacetanilides in refluxing ethyl alcohol in the presence of sodium acetate furnished structurally diverse **I2** analogs **5**.

### Tumor cell proliferation assay

Fifteen human cancer cell lines were tested in our studies. Initial screens used human lung cancer cell A549, human epithelial cervical cancer Hela cells, human prostate cancer PC-3 cells, human epidermoid cancer KB-31 cells, human breast cancer MDA-MB-231 cells, and human pancreatic cancer HPAF. Later, a panel of additional ten tumor cells was tested against I2 (complete names are listed in Table S1 in File S1). These cell lines were obtained commercially from ATCC. The first six cell cultures were maintained in 75 cm^2^ cell culture flasks in which the cells were passaged at 70–80% confluence every 2–3 days. All cancer cells were cultured in RPMI 1640 containing 10% fetal calf serum, 100 U/mL penicillin, 100 µg/mL streptomycin, and 2 mM L-lutamine (complete RPMI medium). All cancer cells were plated at 1×10^4^ cells per well in a 96 well plate. Drugs of different concentrations were then added. MTS assays (CellTiter 96® AQueous One Solution Cell Proliferation Assay, Promega) were performed after 72 h post treatment to determine the cell viability following drug administration.

### In vivo anti-tumor activity

I2 was stored in DMSO at 25 mM at -20 degree. For low dose I2 injection (5 mg/kg), 1 mL solution containing 50 µL I2 stock solution, 50 µL Tween-80, and 900 µL PBS was freshly prepared. This solution was injected once per day at 200 ul per injection. For high dose injection (50 mg/kg), the 25 mM I2 stock was injected directly, with 50 µl per injection twice per day. PC-3 prostate cells were maintained in 75 cm^2^ cell culture flasks in which the cells were passaged at 90% confluence every 4 days. The cells were cultured in Dulbecco's Modified Eagle Medium (DMEM) (Carlsbad, CA) containing 10% FBS, 100 U/mL penicillin, 100 mg/mL streptomycin, and 2 mM L-glutamine (complete medium). Athymic BALB/c nu/nu male mice (6 weeks) were purchased from the Charles River Laboratory, and maintained under pathogen-free conditions. The tumor cell suspension (0.1 ml, 8×10^6^ cells/mL) was injected subcutaneously into the mice. In the tumor growth inhibition experiments, when tumors were palpable at 11 days after inoculation (4–6 mm in diameter), the nude mice were randomly divided into 3 groups, with 4 animals per group. The tumor-bearing mice received intraperitoneal (i.p.) administration of the I2 solution described above at doses of 5 mg/kg and 50 mg/kg. The animals received 5 daily injections as described above per week for 2 weeks. DMSO (50 ul each, twice per day, 5 days per week) injection was used as a control. The body weight and tumor size were recorded 3 times per week. Tumor weight was calculated according to the formula: Tumor volume = 0.5×(length in mm)×(width in mm)×(width in mm).

### Analysis of mitotic inhibition in live zebrafish embryos

A transgenic line of zebrafish expressing zebrafish H2A-GFP in nuclei[40] was used to exam mitotic timing and chromosome segregation using time-lapse video microscopy. H2A-GFP heterozygous fish were in-crossed, and the resulting embryos were grown at 28.5°C. These embryos were dechorinated and treated with I2 at 22 hpf followed by 2–4 h incubation at 28.5°C. Embryos were then placed in low melt agarose along with 150 ug/ml tricaine and submerged in Holtfreter's solution with 5 uM I2. Yolk extension of the I2 and control embryos was imaged. Optical sections (2 µm) of six consecutive images were collected every 2 min for 3 hours and merged on a Zeiss AX10 confocal microscope. Average time to finish one mitotic cell cycle as well as numbers of cells undergoing mitosis per image collection series of the embryo are calculated.

### PLK1 enzyme inhibition assay

Following manufacturer's protocol, an ADP-Glo Kinase Assay (Promega) was used to determine I2 inhibition of PLK1 using ON-01910 as a positive control. ADP-Glo™ Kinase Assay is a luminescent kinase assay that measures ADP formed via kinase reaction; after removing unused ATP, ADP is converted into ATP, which is further converted into light by Ultra-Glo™ Luciferase. The luminescent signal positively correlates kinase activity. Purified PLK1 and its substrate casein protein were used in our assay.

## Supporting Information

File S1 Supporting information.
(DOC)
