# Supplementary material for: A Novel Anti-Tumor Inhibitor Identified by Virtual Screen with PLK1 Structure and Zebrafish Assay
Source: PLoS One. 2013 Apr 26;8(4):e53317. doi: 10.1371/journal.pone.0053317 (PMC3637257; doi:10.1371/journal.pone.0053317)
Supplement: File S1 — Supporting information. (DOC) [file pone.0053317.s001.doc]

**Supplementary materials:**

**Scheme 1.** Chemical synthesis of I2 and its analogs. Conditions and reagents: a) DMF, POCl_3_, reflux; b) NH_2_OH, DMSO, 100^o^C; c) NaHS, EtOH, reflux; d) Bromoacetanilide, NaOAc, EtOH, reflux.

**Table 1.** Additional compounds tested for zebrafish embryonic cleavage inhibition and tumor cell line PC-3

| Entry | CID | Chemical Structure | Vender ID | Effective concentration of inhibiting zebrafish cleavage | IC50 inhibiting PC-3 |
| --- | --- | --- | --- | --- | --- |
| 1  Lead Compound  (I2) | [1450405](http://pubchem.ncbi.nlm.nih.gov/summary/summary.cgi?cid=1450405&loc=ec_rcs) |  | ASN 05588327 | **1μM** | **1μM** |
| **12 Compounds from commercial vender** | | | | |  |
| 2 | [1450551](http://pubchem.ncbi.nlm.nih.gov/summary/summary.cgi?cid=1450551&loc=ec_rcs) |  | ASN 05588521 | **-** | **40μM** |
| 3 | [1164455](http://pubchem.ncbi.nlm.nih.gov/summary/summary.cgi?cid=1164455&loc=ec_rcs) |  | ASN 02254668 | **-** | **40μM** |
| 4 | [3207535](http://www.ncbi.nlm.nih.gov/sites/entrez?cmd=search&db=pccompound&term=3207535%5Buid%5D) |  | ASN 05545607 | **-** | **20μM** |
| 5 | [3208047](http://www.ncbi.nlm.nih.gov/sites/entrez?cmd=search&db=pccompound&term=3208047%5Buid%5D) |  | ASN 05547156 | **-** | **20μM** |
| 6 | [1450453](http://www.ncbi.nlm.nih.gov/sites/entrez?cmd=search&db=pccompound&term=1450453%5Buid%5D) |  | ASN 05588393 | ­**-** | **10μM** |
| 7 | [1156726](http://www.ncbi.nlm.nih.gov/sites/entrez?cmd=search&db=pccompound&term=1156726%5Buid%5D) |  | ASN 02240571 | **-** | **20μM** |
| 8 | [1164478](http://www.ncbi.nlm.nih.gov/sites/entrez?cmd=search&db=pccompound&term=1164478%5Buid%5D) |  | ASN 02254711 | **-** | **10μM** |
| 9  (A3) | [1164459](http://www.ncbi.nlm.nih.gov/sites/entrez?cmd=search&db=pccompound&term=1164459%5Buid%5D) |  | ASN 02254675 | **+**  **(2μM)** | **5μM** |
| 10 | [1450380](http://www.ncbi.nlm.nih.gov/sites/entrez?cmd=search&db=pccompound&term=1450380%5Buid%5D) |  | ASN 05588318 | **-** | **10μM** |
| 11 | [1132102](http://www.ncbi.nlm.nih.gov/sites/entrez?cmd=search&db=pccompound&term=1132102%5Buid%5D) |  | BAS 02539216 | **-** | **10μM** |
| 12 | [1374544](http://www.ncbi.nlm.nih.gov/sites/entrez?cmd=search&db=pccompound&term=1374544%5Buid%5D) |  | ASN 02538812 | **-** | **50μM** |
| 13 | [3171526](http://www.ncbi.nlm.nih.gov/sites/entrez?cmd=search&db=pccompound&term=3171526%5Buid%5D) |  | ASN 03321954 | **-** | **10μM** |
| **20 compounds from chemical synthesis** | | | | |  |
| 14 | **5a** |  | - | **-** | **40μM** |
| 15 | **5b** |  | - | **-** | **10μM** |
| 16 | **5c** |  | - | **-** | **10μM** |
| 17 | **5d** |  | - | **+**  **(50μM)** | **10μM** |
| 18 | **5e** |  | - | **-** | **10μM** |
| 19 | **5f** |  | - | **-** | **10μM** |
| 20 | **5g** |  | - | **-** | **10μM** |
| 21 | **5h** |  | - | **-** | **10μM** |
| 22 | **5i** |  | - | **-** | **20μM** |
| 23 | **5j** |  | - | **-** | **10μM** |
| 24 | **5k** |  | - | **-** | **30μM** |
| 25 | **5l** |  | - | **-** | **10μM** |
| 26 | **5m** |  | - | **-** | **20μM** |
| 27 | **5n** |  | - | **-** | **10μM** |
| 28 | **5o** |  | - | **-** | **20μM** |
| 29 | **5p** |  | - | **-** | **10μM** |
| 30 | **5q** |  | - | **-** | **20μM** |
| 31 | **5r** |  | - | **-** | **40μM** |
| 32 | **5s** |  | - | **-** | **40μM** |
| 33 | **5t** |  |  | **-** | **50μM** |

Table 2. Mitosis inhibition of zebrafish cells by I2

|  | Cell Numbers | |
| --- | --- | --- |
| Experiments | Control | I2 |
| Exp 1 | 139 | 148 |
| Exp 2 | 151 | 146 |
| Exp 3 | 146 | 140 |
| Avg | 145 | 144 |
| Cell undergoing division | 29 | 11 |
| Cell division (%) | 19.95 | 7.60 |
| Time to finish mitosis (min) | 16 | >50 |

**Synthetic methods for I2 and its analogs.**

1. Synthesis of 2-chloro-6-(substituted)quinoline-3-carboxaldehyde (**2)**

A mixture of acetanilide derivative **1** (20 mmol) and phosphorous oxychloride (140 mmol) in dimethyl formamide (50 mmol) was heated at 75 ^o^C for 10 h, cooled to room temperature then poured onto 400 ml ice-water, and the formed precipitate was collected by filteration. The obtained crude product was recrystallized from ethyl acetate to afford the desired producds (**2)**.

2. Synthesis of 2-chloro-6-(substituted)quinoline-3-carbonitrile (**3)**

To a solution of hydroxylamine hydrochloride (18 mmol) in DMSO (20 ml) was added 2-chloro-6-(substituted)quinoline-3-carboxaldehyde **(2)** (10 mmol), and the resulting reaction solution was stirred and heated for 30 min at 100^o^C. After cooling to room temperature, water (100 ml) was added to the reaction mixture, which was then extracted with diethyl ether (5×100 ml). The combined ether layers were washed with water (4×100 ml) and dried over K_2_CO_3_. Removal of the ether by rotary evaporation and high vacuum yielded the corresponding nitrile (**3**).

3. Synthesis of 2-merca-6-(substituted)quinoline-3-carbonitrile (**4)**

A mixture of 2-chloro-6-(substituted)quinoline-3-carbonitrile (**3)** (6 mmol) and sodium hydrosulphide (30% aq. solution 8 ml) in ethyl alcohol (50 ml) was refluxed overnight. The reaction mixture was cooled to room temperature, poured into 100 ml of cold water and acidified with acetic acid. The formed precipitate was filtered, washed with water and cold ethyl alcohol, recrystallized form ethyl alcohol to yield 2-merca-6-(substituted)quinoline-3-carbonitrile (**4).**

4. Synthesis of I2 analogs (**5**)

To a suspension of 2-merca-6-(substituted)quinoline-3-carbonitrile (**4**) (1 mmol) and anhydrous sodium acetate (1.5 mmol) in ethanol (5 ml) was added the appropriate bromoacetanilide (1 mmol). The reaction mixture was refluxed overnight. On cooling, the precipitate was collected and recrystallized from ethanol to deliver desired product (**5**).

**Characterization of synthesized I2 analogs** (**5a-t**)

2-(3-Cyano-6-methoxyquinolin-2-ylthio)-N-(2-methoxyphenyl)acetamide (**5a**), ^1^H NMR (400 MHz, CDCl_3_) *δ* 9.38 (*s*, 1H), 8.35-8.38 (*m*, 2H), 8.01 (*d*, *J* = 9.2 Hz, 1H), 7.50 (*d*, *J* = 8.8 Hz, 1H), 7.09 (*s*, 1H), 6.93-7.03 (*m*, 2H), 6.75 (*d*, *J* = 8.0 Hz, 1H), 4.21 (*s*, 2H), 3.97 (*s*, 3H), 3.44 (*s*, 3H); ^13^C NMR (100 MHz, CDCl_3_) *δ* 166.8, 158.3, 154.5, 148.1, 144.6, 141.7, 129.8, 127.5, 125.8, 125.3, 123.8, 120.9, 120.2, 115.2, 109.9, 106.1, 105.4, 55.8, 55.2, 34.6; HRMS (*m/z*) calc. for C20H18N3O3S (+) 380.1069, found 380.1063.

N-(5-Chloro-2-methoxyphenyl)-2-(3-cyano-6-methoxyquinolin-2-ylthio)acetamide (**5b**), ^1^H NMR (500 MHz, CDCl_3_) *δ* 9.38 (*s*, 1H), 8.43 (*s*, 1H), 8.34 (*s*, 1H), 7.98 (*d*, *J* = 9.2 Hz, 1H), 7.49 (*dd*, *J* = 2.75, 9.2 Hz, 1H), 7.08 (*s*, 1H), 6.95 (*d*, *J* = 8.75 Hz, 1H), 6.64 (*d*, *J* = 8.65 Hz, 1H), 4.18 (*s*, 2H), 3.96 (*s*, 3H), 3.40 (*s*, 3H); ^13^C NMR (125 MHz, DMSO) *δ* 167.5, 158.2, 154.5, 148.2, 144.4, 143.4, 129.6, 128.9, 126.2, 125.6, 124.5, 123.9, 120.5, 116.1, 112.9, 107.2, 105.2, 56.5, 56.3, 34.9; HRMS (*m/z*) calc. for C20H17ClN3O3S (+) 414.0679, found 414.0692.

2-(3-Cyano-6-methoxyquinolin-2-ylthio)-N-(2,5-dimethoxyphenyl)acetamide (**5c**), ^1^H NMR (400 MHz, CDCl_3_) *δ* 9.38 (*s*, 1H), 8.35 (*s*, 1H), 8.11 (*s*, 1H), 8.01 (*d*, *J* = 9.2 Hz, 1H), 7.51 (*d*, *J* = 8.8 Hz, 1H), 7.09 (*s*, 1H), 6.69 (*d*, *J* = 9.2 Hz, 1H), 6.53 (*d*, *J* = 8.8 Hz, 1H), 4.20 (*s*, 2H), 3.97 (*s*, 3H), 3.78 (*s*, 3H), 3.39 (*s*, 3H), ^13^C NMR (125 MHz, DMSO) *δ* 167.1, 158.2, 155.9, 154.4, 153.5, 144.4, 143.4, 129.6, 128.5, 126.2, 125.6, 116.2, 112.3, 108.6, 107.9, 107.2, 105.3, 56.6, 56.3, 55.9, 34.9; HRMS (*m/z*) calc. for C21H20N3O4S (+) 410.1175, found 410.1175.

N-(2-Bromophenyl)-2-(3-cyano-6-methoxyquinolin-2-ylthio)acetamide (**5d**), ^1^H NMR (400 MHz, CDCl_3_) *δ* 9.02 (*s*, 1H), 8.35 (*s*, 1H), 8.29 (*d*, *J* = 8.4 Hz, 1H), 7.98 (*d*, *J* = 9.2 Hz, 1H), 7.48 (*d*, *J* = 9.2 Hz, 1H), 7.43 (*d*, *J* = 8.4 Hz, 1H), 7.31 (*m*, 1H), 7.08 (*s*, 1H), 7.01 (*t*, *J* = 8.4 Hz, 1H), 4.26 (*s*, 2H), 3.96 (*s*, 3H); ^13^C NMR (100 MHz, CDCl_3_) *δ* 166.7, 158.4, 153.6, 144.7, 141.8, 135.6, 132.2, 129.9, 128.2, 125.9, 125.4, 122.5, 115.3, 113.8, 105.9, 105.4, 55.8, 34.5; HRMS (*m/z*) calc. for C19H15BrN3O2S (+) 428.0068, found 428.0070.

N-(2-Chlorophenyl)-2-(3-cyano-6-methoxyquinolin-2-ylthio)acetamide (**5e**), ^1^H NMR (400 MHz, CDCl_3_) *δ* 9.17 (*s*, 1H), 8.35 (*s*, 1H), 8.33 (*d*, *J* = 9.2 Hz, 1H), 7.98 (*d*, *J* = 9.2 Hz, 1H), 7.48 (*d*, *J* = 9.2 Hz, 1H), 7.25-7.28 (*m*, *2*H), 7.08 (*s*, 1H), 7.01 (*t*, *J* = 8.0 Hz, 1H), 4.25 (*s*, 2H), 3.96 (*s*, 3H); ^13^C NMR (125 MHz, CDCl_3_) *δ* 166.9, 158.5, 153.9, 144.7, 141.9, 134.7, 129.8, 129.0, 127.6, 125.9, 125.5, 124.9, 123.3, 122.2, 115.2, 106.1, 105.7, 55.8, 34.6; HRMS (*m/z*) calc. for C19H15ClN3O2S (+) 384.0574, found 384.0577.

2-(3-Cyano-6-methoxyquinolin-2-ylthio)-N-(pyridin-2-yl)acetamide (**5f**), ^1^H NMR (500 MHz, CDCl_3_) *δ* 10.03 (*s*, 1H), 8.32 (*s*, 1H), 8.18-8.21 (*m*, 2H), 8.12 (*d*, *J* = 9.1 Hz, 1H), 7.67 (*t*, *J* = 8.0 Hz, 1H), 7.51 (*dd*, *J* = 2.7, 9.2 Hz, 1H), 7.06 (*d*, *J* = 2.6 Hz, 1H), 6.98 (*dd*, *J* = 4.8, 7.3 Hz, 1H), 4.12 (*s*, 2H), 3.95 (*s*, 3H); ^13^C NMR (100 MHz, CDCl_3_) *δ* 167.3, 158.5, 154.5, 151.4, 147.9, 144.6, 141.8, 138.2, 129.6, 126.2, 125.5, 119.8, 115.2, 113.9, 105.9, 105.4, 55.8, 35.3; HRMS (*m/z*) calc. for C18H15N4O2S (+) 351.0916, found 351.0917.

N-(5-Chloropyridin-2-yl)-2-(3-cyano-6-methoxyquinolin-2-ylthio)acetamide (**5g**), ^1^H NMR (400 MHz, CDCl_3_) *δ* 10.31 (*s*, 1H), 8.36 (*s*, 1H), 8.12-8.21 (*m*, 3H), 7.64 (*d*, *J* = 8.4 Hz, 1H), 7.54 (*t*, *J* = 8.8 Hz, 1H), 7.10 (*s*, 1H), 4.12 (*s*, 2H), 3.98 (*s*, 3H); ^13^C NMR (125 MHz, DMSO) *δ* 167.7, 158.0, 154.6, 151.1, 146.9, 144.4, 143.2, 138.5, 129.4, 126.2, 125.7, 125.5, 116.3, 115.1, 107.2, 105.1, 56.3, 35.3; HRMS (*m/z*) calc. for C18H14ClN4O2S (+) 385.0526, found 385.0528.

2-(3-Cyano-6-methoxyquinolin-2-ylthio)-N-(pyrazin-2-yl)acetamide (**5h**), ^1^H NMR (500 MHz, CDCl_3_) *δ* 10.38 (*s*, 1H), 9.52 (*s*, 1H), 8.35 (*s*, 1H), 8.30 (*d*, *J* = 2.5 Hz, 1H), 8.16 (*d*, *J* = 2.5 Hz, 1H), 8.10 (*d,* *J* = 9.2 Hz, 1H), 7.54 (*dd,* *J* = 2.8, 9.2 Hz, 1H), 7.09 (*d,* *J* = 2.7 Hz, 1H), 4.13 (*s*, 2H), 3.96 (*s*, 3H); ^13^C NMR (125 MHz, DMSO) *δ* 167.9, 158.1, 154.5, 149.2, 144.4, 143.2, 140.4, 136.7, 129.4, 126.2, 125.5, 116.3, 107.3, 105.1, 56.3, 35.1; HRMS (*m/z*) calc. for C17H14N5O2S (+) 352.0868, found 352.0877.

N-(2,5-Bis(trifluoromethyl)phenyl)-2-(3-cyano-6-methoxyquinolin-2-ylthio)acetamide (**5i**), ^1^H NMR (400 MHz, CDCl_3_) *δ* 9.01 (*s*, 1H), 8.53 (*s*, 1H), 8.35 (*s*, 1H), 7.87 (*d*, *J* = 9.2 Hz, 1H), 7.62 (*d*, *J* = 8.4 Hz, 1H), 7.43-7.48 (*m*, 2H), 7.06 (*d,* *J* = 2.8 Hz, 1H), 4.23 (*s*, 2H), 3.94 (*s*, 3H); ^13^C NMR (125 MHz, CDCl_3_) *δ* 167.1, 158.6, 153.2, 144.7, 141.9, 135.9, 129.7, 126.7, 126.1, 125.6, 124.1, 121.9, 121.8, 121.3, 115.1, 106.0, 105.6, 55.8, 34.4; HRMS (*m/z*) calc. for C21H14F6N3O2S (+) 486.0711, found 486.0682.

2-(3-Cyano-6-methoxyquinolin-2-ylthio)-N-(thiazol-2-yl)acetamide (**5j**), ^1^H NMR (400 MHz, DMSO-d6) *δ* 12.54 (*s*, 1H), 8.84 (*s*, 1H), 7.66 (*d*, *J* = 9.2 Hz, 1H), 7.48-7.53 (*m*, 2H), 7.39 (*d,* *J* = 2.4 Hz, 1H), 7.18 (*d,* *J* = 3.2 Hz, 1H), 4.36 (*s*, 2H), 3.87 (*s*, 3H); ^13^C NMR (125 MHz, DMSO) *δ* 167.1, 158.1, 154.3, 144.4, 143.2, 138.3, 129.3, 126.2, 125.5, 116.2, 114.1, 107.3, 105.1, 56.3, 34.2; HRMS (*m/z*) calc. for C16H13N4O2S2 (+) 357.0480, found 357.0475.

2-(3-Cyano-6-methoxyquinolin-2-ylthio)-N-(2-hydroxyphenyl)acetamide (**5k**), ^1^H NMR (400 MHz, CDCl_3_) *δ* 9.84 (*s*, 1H), 8.58 (*s*, 1H), 8.37 (*s*, 1H), 7.93 (*d*, *J* = 8.8 Hz, 1H), 7.52 (*dd*, *J* = 3.2, 9.2 Hz, 1H), 7.05-7.11 (*m*, 2H), 6.97 (*d,* *J* = 6.8 Hz, 1H), 6.80 (*d,* *J* = 8.0 Hz, 1H), 6.75 (*d,* *J* = 6.8 Hz, 1H), 4.17 (*s*, 2H), 3.96 (*s*, 3H); ^13^C NMR (125 MHz, DMSO) *δ* 166.9, 158.1, 154.5, 147.7, 144.5, 143.3, 129.7, 126.8, 126.1, 125.6, 124.8, 121.7, 119.5, 116.2, 115.7, 107.1, 105.2, 56.3, 34.9; HRMS (*m/z*) calc. for C19H16N3O3S (+) 366.0912, found 366.0900.

2-(3-Cyano-6-methoxyquinolin-2-ylthio)-N-(2,4-dichlorophenyl)acetamide (**5l**), ^1^H NMR (400 MHz, CDCl_3_) *δ* 9.17 (*s*, 1H), 8.34 (*s*, 1H), 8.29 (*d*, *J* = 8.8 Hz, 1H), 7.94 (*d*, *J* = 8.8 Hz, 1H), 7.46 (*dd*, *J* = 2.8, 9.2 Hz, 1H), 7.20-7.26 (*m*, 2H), 7.06 (*d,* *J* = 2.4 Hz, 1H), 4.21 (*s*, 2H), 3.96 (*s*, 3H); ^13^C NMR (100 MHz, DMSO) *δ* 167.1, 158.4, 153.9, 144.6, 141.9, 133.4, 129.6, 129.3, 128.6, 127.8, 126.1, 125.5, 123.7, 122.7, 115.1, 106.0, 105.6, 55.8, 34.5; HRMS (*m/z*) calc. for C19H14Cl2N3O2S (+) 418.0184, found 418.0164.

2-(3-Cyano-6-methoxyquinolin-2-ylthio)-N-(2,6-diethylphenyl)acetamide (**5m**), ^1^H NMR (400 MHz, CDCl_3_) *δ* 8.43 (*s*, 1H), 8.37 (*s*, 1H), 7.90 (*d*, *J* = 9.2 Hz, 1H), 7.49 (*dd*, *J* = 2.8, 9.2 Hz, 1H), 7.19 (*t,* *J* = 7.6 Hz, 1H), 7.09 (*d*, *J* = 2.8 Hz, 1H), 7.06 (*d*, *J* = 7.6 Hz, 1H), 4.24 (*s*, 2H), 3.96 (*s*, 3H), 2.46 (*q*, *J* = 7.2 Hz, 4H), 0.98 (*t*, *J* = 7.2 Hz, 6H); ^13^C NMR (100 MHz, DMSO) *δ* 166.8, 157.9, 154.6, 144.5, 143.1, 141.9, 134.2, 129.5, 127.6, 126.3, 125.9, 125.5, 116.3, 107.1, 105.3, 56.2, 33.9, 24.6, 14.9; HRMS (*m/z*) calc. for C23H24N3O2S (+) 406.1589, found 406.1585.

2-(3-Cano-6-methoxyquinolin-2-ylthio)-N-(4-(dimethylamino)phenyl)acetamide (**5n**), ^1^H NMR (400 MHz, CDCl_3_) *δ* 9.17 (*s*, 1H), 8.34 (*s*, 1H), 7.94 (*d*, *J* = 9.2 Hz, 1H), 7.51 (*dd*, *J* = 2.8, 9.2 Hz, 1H), 7.29 (*d*, *J* = 9.2 Hz, 2H), 7.08 (*d,* *J* = 2.8 Hz, 1H), 6.62 (*d*, *J* = 9.2 Hz, 2H), 4.09 (*s*, 2H), 3.96 (*s*, 3H), 2.88 (*s*, 6H); ^13^C NMR (125 MHz, DMSO) *δ* 165.5, 157.9, 154.9, 147.7, 144.5, 143.1, 129.5, 129.3, 126.1, 125.4, 121.2, 116.3, 113.2, 107.2, 105.2, 56.3, 40.9, 35.4; HRMS (*m/z*) calc. for C21H21N4O2S (+) 393.1385, found 393.1385.

2-(6-Chloro-3-cyanoquinolin-2-ylthio)-N-(2-methoxy-5-methylphenyl)acetamide (**5o**), ^1^H NMR (400 MHz, CDCl_3_) *δ* 9.15 (*s*, 1H), 8.35 (*s*, 1H), 8.18 (*d*, *J* = 2.0 Hz, 1H), 8.02 (*d*, *J* =8.8 Hz, 1H), 7.82 (*d*, *J* = 2.4Hz, 1H), 7.77 (*dd*, *J* = 2.4, 9.2 Hz, 1H), 6.78 (*dd*, *J* = 1.6, 8.4 Hz, 1H), 6.64 (*d,* *J* = 8.4 Hz, 1H), 4.20 (*s*, 2H), 3.46 (*s*, 3H), 2.28 (*s*, 3H); ^13^C NMR (100 MHz, CDCl_3_) *δ* 166.1, 157.9, 146.6, 145.9, 141.9, 133.9, 133.2, 130.1, 129.9, 127.0, 126.7, 124.7, 124.2, 120.8, 114.6, 109.8, 106.9, 55.4, 34.8, 20.9; HRMS (*m/z*) calc. for C20H17ClN3O2S (+) 398.0730, found 398.0710.

2-(6-Chloro-3-cyanoquinolin-2-ylthio)-N-(2-(trifluoromethyl)phenyl)acetamide (**5p**), ^1^H NMR (400 MHz, CDCl_3_) *δ* 8.75 (*s*, 1H), 8.37 (*s*, 1H), 8.10 (*d*, *J* = 7.6 Hz, 1H), 7.92 (*d*, *J* = 9.2 Hz, 1H), 7.81 (*s*, 1H), 7.74 (*d*, *J* = 7.2 Hz, 1H), 7.52 (*t*, *J* = 8.0 Hz, 2H), 7.23 (*d,* *J* = 8.0 Hz, 1H), 4.24 (*s*, 2H); ^13^C NMR (100 MHz, CDCl_3_) *δ* 166.5, 156.8, 146.7, 142.1, 134.7, 134.2, 133.4, 132.8, 129.8, 126.7, 126.0, 125.9, 125.0, 124.9, 124.8, 114.6, 106.8, 34.2; HRMS (*m/z*) calc. for C19H12ClF3N3OS (+) 422.0342, found 422.0324.

2-(3-Cyano-6-methylquinolin-2-ylthio)-N-(2-methoxy-5-methylphenyl)acetamide (**5q**), ^1^H NMR (400 MHz, CDCl_3_) *δ* 9.31 (*s*, 1H), 8.35 (*s*, 1H), 8.18 (*d*, *J* = 2.0 Hz, 1H), 7.98 (*d*, *J* = 8.4 Hz, 1H), 7.69 (*dd*, *J* = 2.0, 8.8 Hz, 1H), 7.60 (*s*, 1H), 6.77 (*dd,* *J* = 1.6, 8.4 Hz, 1H), 6.62 (*d*, *J* = 8.0 Hz, 1H), 4.20 (*s*, 2H), 3.38 (*s*, 3H), 2.56 (*s*, 3H), 2.28 (*s*, 3H); ^13^C NMR (100 MHz, CDCl_3_) *δ* 166.7, 156.3, 146.9, 146.1, 142.5, 137.6, 135.4, 130.5, 128.1, 127.2, 126.9, 124.3, 124.1, 120.8, 115.2, 109.8, 105.8, 55.3, 34.6, 21.4, 20.9; HRMS (*m/z*) calc. for C21H20N3O2S (+) 378.1276, found 378.1282.

2-(3-Cyano-6-methylquinolin-2-ylthio)-N-(2-(trifluoromethyl)phenyl)acetamide (**5r**), ^1^H NMR (400 MHz, CDCl_3_) *δ* 8.89 (*s*, 1H), 8.37 (*s*, 1H), 8.09 (*d*, *J* = 8.0 Hz, 1H), 7.88 (*d*, *J* = 8.4 Hz, 1H), 7.64 (*dd*, *J* = 2.0, 8.8 Hz, 1H), 7.58 (*s*, 1H), 7.50-7.55 (*m*, 2H), 7.22 (*t,* *J* = 8.0 Hz, 1H), 4.24 (*s*, 2H), 2.54 (*s*, 3H); ^13^C NMR (125 MHz, CDCl_3_) *δ* 167.0, 155.3, 147.1, 142.7, 137.8, 135.7, 134.9, 132.7, 128.0, 126.9, 126.0, 125.9, 125.3, 124.8, 124.4, 120.9, 115.2, 105.7, 34.2, 21.4; HRMS (*m/z*) calc. for C20H15F3N3OS (+) 402.0888, found 402.0889.

2-(6-Bromo-3-cyanoquinolin-2-ylthio)-N-(2-methoxy-5-methylphenyl)acetamide (**5s**), ^1^H NMR (400 MHz, CDCl_3_) *δ* 9.14 (*s*, 1H), 8.34 (*s*, 1H), 8.18 (*d*, *J* = 1.6 Hz, 1H), 7.89-8.0 (*m*, 3H), 6.79 (*d*, *J* = 8.4 Hz, 1H), 6.64 (*d*, *J* = 8.0 Hz, 1H), 4.20 (*s*, 2H), 3.45 (*s*, 3H), 2.28 (*s*, 3H); ^13^C NMR (100 MHz, CDCl_3_) *δ* 166.1, 158.1, 146.8, 145.9, 141.9, 136.5, 130.6, 130.0, 129.9, 127.0, 125.2, 124.2, 121.1, 120.8, 114.6, 109.8, 106.9, 55.4, 34.8, 20.9; HRMS (*m/z*) calc. for C20H17BrN3O2S (+) 442.0225, found 442.0220.

2-(6-Bromo-3-cyanoquinolin-2-ylthio)-N-(2-(trifluoromethyl)phenyl)acetamide (**5t**), ^1^H NMR (400 MHz, CDCl_3_) *δ* 8.75 (*s*, 1H), 8.37 (*s*, 1H), 8.10 (*d*, *J* = 8.0 Hz, 1H), 7.98 (*d*, *J* = 1.6 Hz, 1H), 7.87 (*m*, 2H), 7.51-7.56 (*m*, 2H), 7.21 (*t,* *J* = 7.6 Hz, 1H), 4.24 (*s*, 2H); ^13^C NMR (100 MHz, CDCl_3_) *δ* 166.5, 156.9, 146.9, 141.9,0 136.7, 134.7, 132.8, 130.0, 129.8, 126.0, 125.9, 125.3, 125.0, 124.9, 122.3, 121.3, 114.6, 106.7, 34.2; HRMS (*m/z*) calc. for C19H12BrF3N3OS (+) 465.9837, found 465.9824.

**Inhibition of additional tumor cells by I2 in vitro and in vivo.**

Ten cancer cell lines were used to test I2 *in vitro*. They were human colon cancer (HCT-8, HCT116, COLO-205 and SW480), pancreatic cancer (MIA Paca-2, PANC-1 BxPC-3 and JF-305), breast cancer (MCF-7) and melanoma (B16). All cell cultures were maintained in 75 cm^2^ cell culture flasks in which the cells were passaged at 70-80% confluence every 2-3 days. HCT-8, SW480, BxPC-3 were cultured with PRIM-1640 (HyClone, SH30809) containing 10% fetal calf serum (FBS), the other cells were cultured with DMEM (HyClone, SH30243) with 10% FBS. All cancer cells were plated at 1x10^4^ cells per well in a 96 well plate. Different concentrations of I2 were then added. MTS assays (CellTiter 96® AQueous One Solution Cell Proliferation Assay, Promega) were performed after 48 h post treatment to determine the cell viability following drug administration.


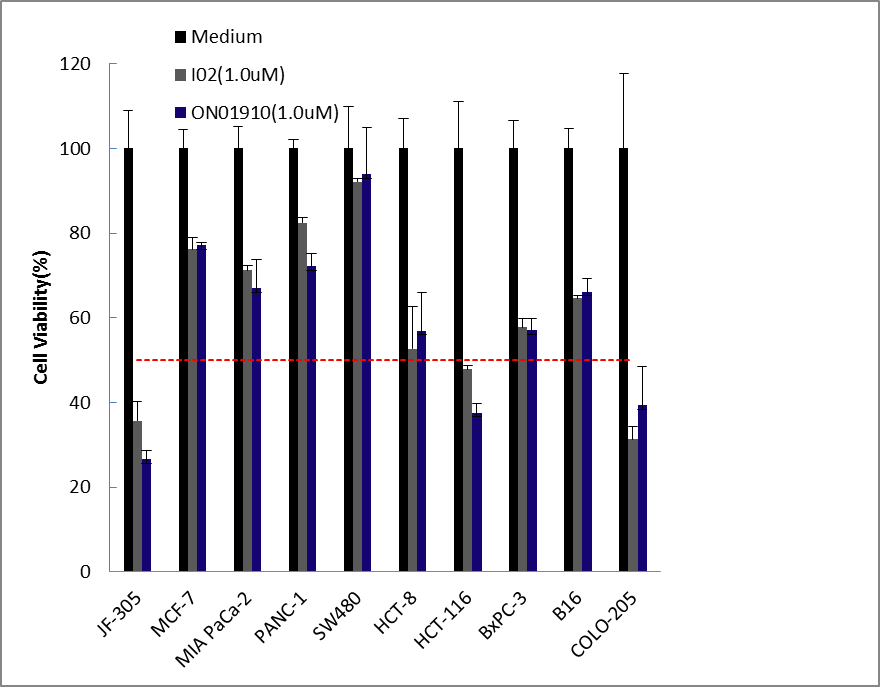


Figure S1. Cell viability after treatment with I2 and ON01910.

For HCT116 tumor xenograft experiment, low dose I2 injection (5 mg/kg) was done in solution containing 50 µL I2 stock solution (10mg/ml in DMSO), 50 µL Tween-80, and 900 µL PBS. This solution was injected once per day at ~200ul per injection. For high dose injection (25 mg/kg), ~100 µl of 10mg/ml in I2 stock in DMSO was injected per day. The animals received 15 daily injections. Injection of 200 µL DMSO:Tween-80:PBS (5%:5%:90%) was used as a control. HCT116 was cultured, prepared for injection and analyzed in mice as that of PC3. Each experimental group includes 8 mice.


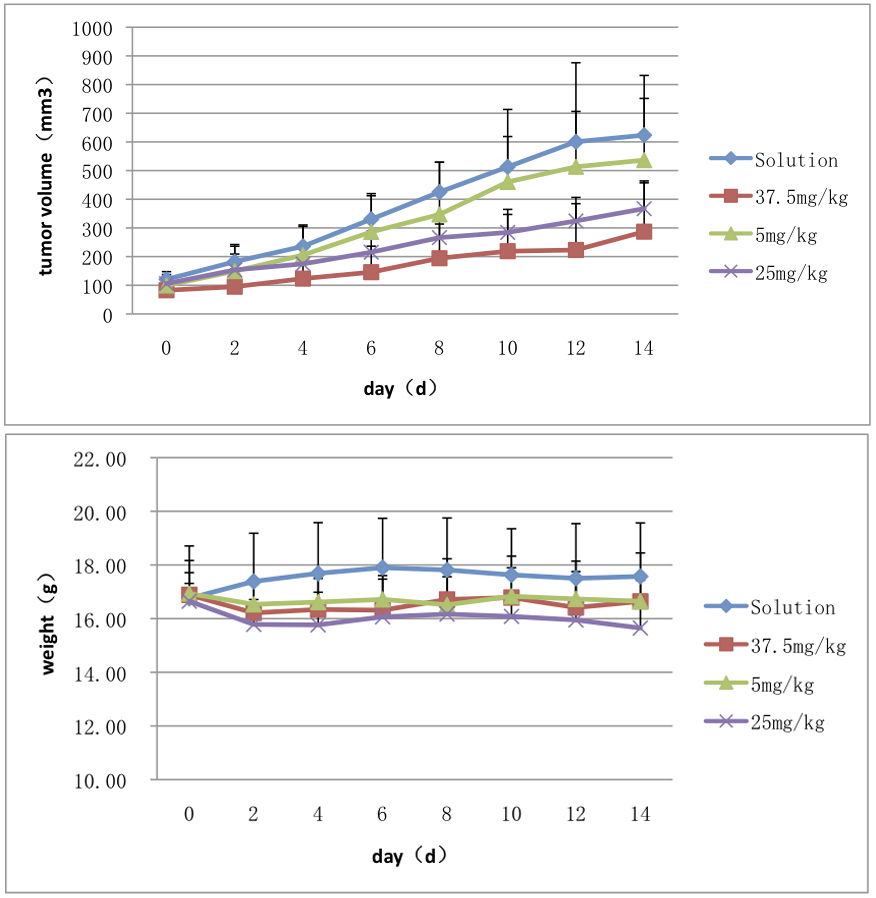


Figure S2. Body weight change and tumor growth in human HC6-116 xenograft after I2 treatment


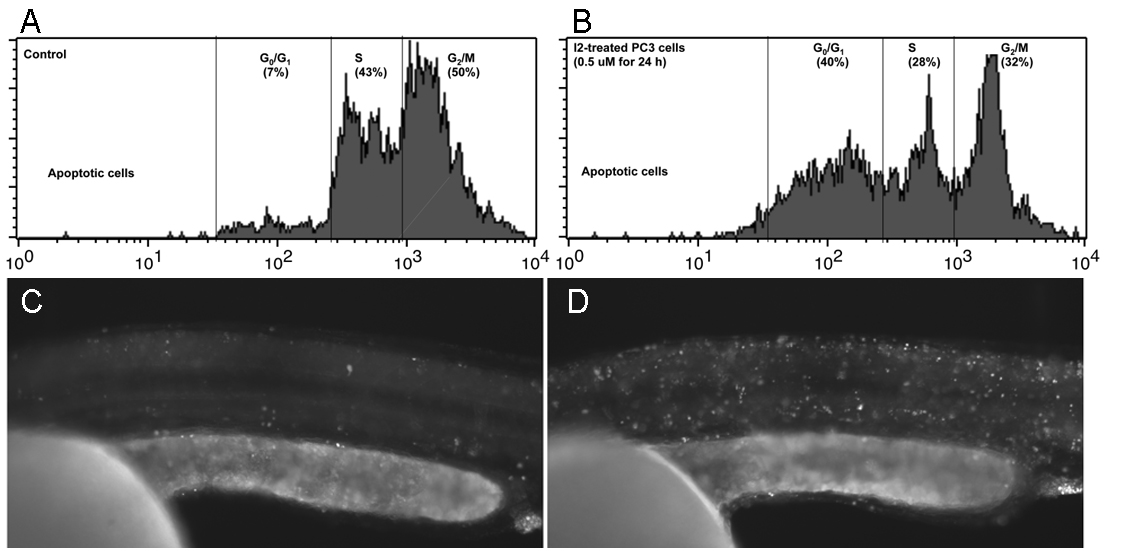


Figure S3. Cell cycle and apopotosis analysis in I2 treated cells and zebrafish embryo. A. PC-3 cells treated with DMSO and B. PC-3 cells treated with 0.5uM I2. C. Zebrafish embryo treated with DMSO and D. treated with I2. Bright fluorescence represents apopototic cells. To conduct cell cycle analysis by propidium iodide (PI) staining in I2-treated PC-3 cells, PC-3 cells were treated with I2 at dose of 0.5 uM for 24 hours, using PBS as control. Treated cells were trypsinized, suspended in complete DMEM medium, centrifuged at 1,500 rpm for 5 min, and the pellet was suspended in PBS. After fixing the cells in 4% paraformaldehyde for 16 hours at 4 °C, the cells were transferred into PBS containing PI (50 µg/ml), RNase A (0.1 mg/ml) and Triton X-100 (0.05%, w/w). After incubation for 60 min at 37°C, the cells were collected down by centrifuge and washed by PBS for another time. Cells were analyzed by SCAN flow cytometer (Becton Dickinson, Mountain View, CA) using mean FL-2 to assess PI fluorescence.

Zebrafish embryos were treated with I2 (5uM) from late gastrulation stage and stained with acridine orange to detect apoptosis in living embryos at 24 hpf. Acridine orange was dissolved in Holtreter’s buffer (5 mg/ml) and embryos were stained at 5 ug/ml for 30 min in Holtreter’s buffer followed by washing embryos three times with the same buffer for 5 min each and imaging under a fluorescence microscope.
